# Pancreatic Cystic Lesions in the Older Patient: A Review of Clinical Guidelines and Management

**DOI:** 10.1007/s11894-025-01004-3

**Published:** 2025-11-11

**Authors:** Sagar Shah, V. Raman Muthusamy

**Affiliations:** Vatche and Tamar Manoukian Division of Digestive Diseases, University of California, Los Angeles, California USA

**Keywords:** Pancreatic cystic lesion, Intraductal papillary mucinous neoplasms, Geriatric

## Abstract

**Purpose of Review:**

The purpose of this review is to summarize key features of the epidemiology of pancreatic cystic lesions (PCLs), highlight the most common PCLs encountered in clinical practice, review relevant guideline recommendations, and briefly introduce innovative new diagnostic and therapeutic technologies in the field, particularly as they relate to the care of elderly patients.

**Recent Findings:**

While CT and MRI are mainstays of diagnostic studies for PCLs, endoscopic ultrasound with fine-needle aspiration and cyst fluid analysis can considerably improve the accuracy of presurgical diagnosis. Surgical interventions remain associated with considerable morbidity. While surveillance of certain lesions is appropriate, surveillance is associated with considerable monetary costs and can cause undue anxiety for patients. There remains uncertainty regarding the optimal management strategy of PCLs given the lack of high-quality evidence to guide recommendations.

**Summary:**

Management decisions for PCLs, be it for surveillance, surgical resection, or invasive diagnostics, should be highly personalized and based on the unique risk-benefit analysis for a given lesion. Especially in elderly populations, physicians should have informed conversations with patients regarding the likelihood of a given PCL meaningfully affecting quality-of-life or life-expectancy.

## Introduction

The incidental detection of pancreatic cystic lesions (PCLs) has increased as high-quality cross-sectional imaging has become more readily available. This, combined with the fact that the incidence of PCLs is correlated with age, has led to a surge of interest in the optimal management strategies for these lesions in our aging population. Autopsy studies that allow for the identification of diminutive, clinically insignificant pancreatic cysts suggest a prevalence as high as 25% [1]. A number of studies evaluating the prevalence of incidental pancreatic cysts seen on CT and MRI studies have estimated prevalence ranging from 2–15% [2–4], with one large meta-analysis suggesting a pooled prevalence of 8% [5]. Studies characterizing the rise in prevalence of these lesions overtime have shown a correlation between evolution of cross-sectional imaging hardware and software and increased detection of pancreatic cysts [6]. The heterogeneity in estimates of incidence and prevalence throughout the literature can, in part, also be attributed to variation in prevalence and incidence based on age – the specific demographic makeup of a given study population considerably affects estimates. The management of pancreatic cysts is particularly relevant to geriatric patients given that the preponderance of cysts are identified in older patients. The purpose of this review is to summarize key features of the epidemiology of PCLs, highlight the most common PCLs encountered in clinical practice, review relevant guideline recommendations, and briefly introduce innovative new diagnostic and therapeutic technologies in the field, particularly as they relate to the care of elderly patients.

## Epidemiology of Pancreatic Cystic Lesions

A study analyzing the incidence of PCLs based on an administrative claims database with over 200 million patients found that between 2010 and 2017, among patients 45 years or older, the annual cumulative incidence of cysts nearly doubled from 6.3 to 11.4 patients per 10,000 (an annual increase of 8.7%/year). During that time, the proportion of patients receiving cross-sectional abdominal imaging increased from only 8.0% to 9.4% (an annual change of 2.6%/year). The authors suggested that based on the data, the increased utilization of imaging accounted for only 31% of the observed increase in cyst incidence [7]. As such, the rise in cyst incidence, if not related solely to increased detection, may be a result of the population becoming more prone to PCLs with age. The association between age and PCLs has been well documented. A recent study evaluating the prevalence of PCLs in all patients undergoing MRI of the abdomen and pelvis at a single, tertiary medical center found the prevalence was roughly 1 in 5 in patients aged 50–59, 1 in 4 in patients aged 60–69, 1 in 3 in patients aged 70–79, and 1 in 2 in patients aged 80–89 [8]. Despite the prevalence of PCLs, the risk of progression of PCLs to invasive cancer is low – on the order of 0.2% per year [9]. Given geriatric patients with pancreatic cysts often have several comorbidities and the overall risk of malignancy is low, risk stratification and appropriate patient selection for surveillance and resection are critically important in these cohorts.

**Fig. 1:**
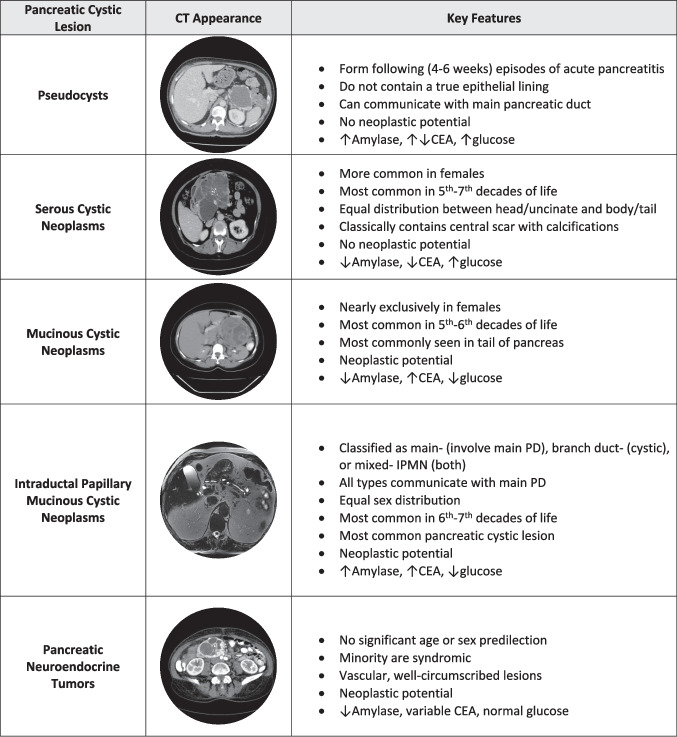
Major classes of pancreatic cystic lesions with summary of major clinical and demographic characteristics

PCLs can broadly be divided into mucinous and non-mucinous cystic lesions (Fig. 1). Mucinous lesions are associated with a risk of malignant transformation while the natural course of non-mucinous lesions is generally benign. The majority of mucinous PCLs are mucinous cystic neoplasms (MCNs) and intraductal papillary mucinous neoplasms (IPMNs). IPMNs are much more commonly encountered in clinical practice than MCNs. The most common non-mucinous lesions include pseudocysts, serous cystic neoplasms (SCNs), solid pseudopapillary neoplasms (SPNs), and cystic neuroendocrine tumors (cNETs). While each of these can be diagnosed based on a combination of clinical, demographic, imaging and fluid characteristics, there are inherent limitations to our ability to accurately classify pancreatic cysts. de Pretis et al. found that there was discrepancy between the pre- and post-operative diagnosis of pancreatic cyst type in a third of patients. More importantly, the authors found that almost 15% of pancreatic cyst resections were performed for asymptomatic benign cysts that were pre-surgically diagnosed as pre-malignant lesions [10]. Thus, the combination of diagnostics (i.e. imaging and cyst fluid analysis) via Endoscopic Ultrasound and fine needle aspiration to obtain cyst fluid for testing is important for diagnostic accuracy and optimizing patient management.

## Cyst Types

### Pseudocysts

Radiologically, pseudocysts appear as well-circumscribed, round peripancreatic fluid collections, most commonly extending from the pancreatic parenchyma into the lesser sac. They typically exhibit homogeneously low attenuation with a well-defined enhancing wall. These lesions can communicate with the main pancreatic duct (MPD). Though high-quality epidemiologic data on this type of PCL are scarce, these cysts are tightly linked to the incidence of acute pancreatitis. Approximately 5% of patients with acute pancreatitis ultimately develop pseudocysts [11]. In one multi-center cohort study including 521 patients with chronic pancreatitis from 13 centers in the United States, pseudocysts were more commonly encountered in male patients; the average age of these patients was not reported[12]. Some retrospective data also suggest that the risk of mortality associated with pseudocysts is higher in elderly patients (≥ 65 years), estimating the mortality risk as 4.5 times that in adults < 65 years old [13]. Although pseudocysts are the most likely lesion for patients with PCLs found as sequelae in the context of pancreatitis, one should also consider the possibility of a mucinous lesion causing an episode of acute pancreatitis.

### Serous Cystic Neoplasms

Pancreatic SCNs are most often seen in women in the fifth to seventh decade of life. The average age of presentation in men is slightly older, potentially related to delays in diagnosis [14]. Radiologically, most SCNs have ‘microcystic’ morphology, often said to have a honeycomb-like appearance. Macrocystic variants have been described, and many SCNs have mixed morphologies with features of both variants [15]. The pathognomonic fibrous, central scar with calcifications can give these lesions a ‘sunburst’ appearance; however, these findings are present on CT or MRI in only a third of cases [3]. Histologically, these lesions are characterized by a single layer of non-ciliated, cuboidal epithelium resembling ovarian surface epithelium. These lesions are generally managed conservatively, with surgical resection reserved for symptomatic cases or when there is diagnostic uncertainty. This poses a unique challenge in geriatric patients, given the higher likelihood of comorbid conditions and neoplastic pancreatic lesions with imaging features that can overlap with SCNs. The growth rate of SCNs is generally slow and these lesions are considered benign as the risk of malignant transformation is exceedingly low, although this has been described [9, 16].

### Mucinous Cystic Neoplasms

MCNs are considered neoplastic precursor lesions of pancreatic adenocarcinoma. They are most commonly diagnosed in the fifth and sixth decades of life, nearly exclusively in women [17]. These lesions are most commonly seen in the tail of the pancreas. MCNs are generally unilocular or septated cysts with a fibrous capsule. The epithelial lining is composed of mucin-producing cells, often with a papillary morphology. MCNs are associated with a characteristic ovarian-type stroma. While these lesions make up a majority of tumors in surgical series [18], smaller MCNs without concerning features on imaging can reasonably be surveilled without surgical intervention [19, 20]. The prognosis of MCNs depends on whether lesions harbor invasive adenocarcinoma or not. Surgical series, likely enriched with higher-risk lesions, have found nearly a third of resected MCNs demonstrate invasive cancer. Data on the association between age and risk of malignancy are conflicting. A large multi-center retrospective cohort study published in *JAMA* did not find age to be independently associated with the risk of malignancy in patients with MCNs [21]; smaller retrospective studies, however, have found associations between age and malignant risk [22]. The prognosis of patients with cancer arising in MCNs is similar to those with conventional pancreatic ductal adenocarcinoma [23].

### Intraductal Papillary Mucinous Neoplasms

IPMNs are characterized by a proliferative epithelium within the pancreatic ductal system associated with the overproduction of mucin. IPMNs are divided into main-duct IPMN with dilation of main pancreatic duct (MPD) and no cystic dilations, branch-duct IPMN (BD-IPMN) composed of cysts that communicate with a non-dilated MPD, and mixed-type that harbor features of both [24]. IPMN lesions involving the main duct classically produce a patulous ampulla exuding mucin, though this “fish mouth” finding is documented in a minority of cases. Regardless of subtype, communication of these lesions with the MPD is a defining feature. Identification of such communication can be difficult, contributing to the difficulty differentiating these lesions from other neoplastic (MCNs) and benign lesions (serous cystadenomas). These lesions are particularly relevant to elderly patients, as they are found most often in the sixth to seventh decades of life. Whereas pseudocysts were historically thought to be most common cyst, the increased utilization of cross-sectional imaging has led to a dramatic rise in the detection of incidental pancreatic cysts in patients without any history of pancreatitis; these lesions are most often presumed to be BD-IPMN [25]. Microscopically, the proliferative epithelium lining the involved ducts can be divided into non-invasive and invasive neoplasms. Non-invasive IPMNs are graded based on the greatest degree of dysplasia, either low‐grade or high‐grade dysplasia/carcinoma in situ. Invasive IPMNs occur in a variety of histologic subtypes (i.e. colloid, tubular) associated with variable clinicopathologic significance [26].

### Less Common Pancreatic Cystic Lesions

Cystic pancreatic neuroendocrine tumors (PNETs) make up roughly 8% of resected PCLs and 15% of all resected PNET [27]. These are rare tumors, with an overall incidence of 0.001%, accounting for 1–2% of all pancreatic neoplasms when not solely accounting for those resected [28]. There does not appear to be a gender predilection. These lesions are most often diagnosed in the sixth decade [29]. These lesions can be associated with symptoms of excess hormone production, though “syndromic” lesions occur in only 30% of cases [30]. Neuroendocrine tumors of the pancreas tend to be highly vascular, well-circumscribed lesions. When large they can produce mass-effect on adjacent structures. As tumors degenerate, they become increasingly cystic, hypervascularity becomes confined to the rim of the lesion, and can sometimes contain calcifications. Recent data suggests that, while functional tumors should be resected, small, non-syndromic tumors can be safely surveilled [31, 32]. Solid pseudopapillary neoplasms are a very uncommon lesion that are nearly exclusively found in women (> 90%), with a median age of presentation between 30 and 38 years [33]. Though most SPNs are benign (even with high-risk histologic features or nodal involvement), given their neoplastic potential (~ 15%) these lesions are most often managed with surgical resection given that they occur in younger patients [34]. These lesions are rarely encountered in geriatric patients.

## Diagnostic Evaluation of Cysts

With rapid improvements in the spatial resolution of cross-sectional imaging, CT and MRI have become mainstays in the evaluation of PCLs. The overarching purpose of diagnostic studies for PCLs is to: 1) make a specific diagnosis and 2) characterize the neoplastic potential of the lesion. While certain pathognomonic features can be seen on CT and MRI facilitating classification of lesions, this occurs in a minority of lesions and significant overlap exists between imaging features. When lesions have suspicious characteristics or a definitive diagnosis cannot be made, EUS can be used to obtain further information.

### CT and MRI

According to the American College of Gastroenterology (ACG) guidelines, MRI is preferred over contrast-enhanced CT for the initial characterization of PCLs given better test characteristics for the identification of IPMNs. The sensitivity and specificity of CT for distinguishing IPMN from other PCLs is 80.6% and 86.4% compared with 96.8% and 90.8% for MRI [35, 36]. A major benefit of MRI with MRCP compared to contrast enhanced CT is its superior ability to delineate communication of PCLs with the pancreatic duct (PD). The sensitivity of MRCP for demonstrating communication of a cyst with the PD has been reported to be as high as 100% [37]. Furthermore, MRI offers an additional important advantage in that it does not expose patients to repeated radiation over the course of surveillance periods. In large meta-analyses evaluating the accuracy of MRI compared to contrast enhanced CT to achieve a specific PCL diagnosis and to differentiate between benign and neoplastic lesions, MRI offers modest advantages in both regards [38].

### Endoscopic Ultrasound

Despite the utility of CT and MRI for the gross characterization of pancreatic cysts, accuracy of these modalities in distinguishing benign from premalignant/malignant varies considerably – estimates range from 57 to 91% [39, 40]. EUS allows for the detailed examination of PCLs and the identification of features characteristic of particular PCL classes. For example, multiple small compartments seen within a PCL on EUS is highly suggestive of an SCN [41]. A cystic lesion without septations or solid components associated with parenchymal calcifications and atrophy is highly suggestive of a pseudocyst. EUS appears to be most helpful in guiding management by improving the detection of true mural nodules, more accurately delineating cyst communication with the main pancreatic duct, and providing cyst fluid for biochemical and cytologic analysis. The differentiation of contrast-enhancing mural nodules from non-enhancing mucin plugs can be challenging. Fujita et al. found that CT and MRI can detect mural nodules in 86% and 71% of cases, respectively. Though contrast-enhanced EUS is not currently approved for use in the United States, this imaging modality was able to correctly identify mural nodules in 100% of cases in one study [42]. Similar results have been demonstrated for the use of EUS for the delineation of main PD dilation. The accuracy of EUS for identification of main PD dilation (using surgical specimen as reference) was 84.9%, compared to 71.7% for CT [43]. Small retrospective studies, however, suggest EUS and MRI with MRCP may be equally capable of assessing cyst communication with the PD [44]. While EUS can be helpful for identification of features associated with high-grade dysplasia (HGD) and malignancy, the quality of images obtained is operator-dependent and interpretation is subject to considerable inter-observer variability [45–47]. The analysis of CEA, amylase, and glucose levels are simple means of obtaining additional diagnostic information using cyst fluid obtained via EUS-FNA. CEA is particularly useful in identifying mucinous lesions. In pooled analyses, elevated CEA was found to have a sensitivity of 63% and specificity of 93% for identifying IPMNs and MCNs. Though CEA levels were once considered the optimal study for the identification of mucinous lesions, test characteristics of cyst fluid glucose levels may be superior. Low glucose levels (< 50 mg/dL) achieve 90% accuracy in distinguishing mucinous from non-mucinous cysts [48]. Elevated amylase is a proxy for communication of a cyst with the MPD and can be seen in pseudocysts and IPMNs. Next-generation sequencing for the identification of cell-free DNA is an emerging technology that can augment characterization of pancreatic cysts. In particular, the *KRAS* and *GNAS* mutations are useful in differentiating MCN’s and IPMN’s [49–51]. The analysis of certain non-*KRAS*/*GNAS* variants can add to the identification of PC with advanced neoplasia (high-grade dysplasia [HGD] or invasive adenocarcinoma). In a study performed Rosenbaum et al. *GNAS* mutations were found to be 100% specific for mucinous neoplasms and the presence of *TP53*, *SMAD4*, and *CDKN2A* variants were exclusively associated with carcinoma [52]. While this new technology is promising, adoption of these techniques is generally limited to tertiary centers given cost and limited insurance coverage. Despite advances in EUS techniques for PCL classification, studies evaluating the pre-surgical diagnostic accuracy of imaging modalities have found that in one-third of the cases, pre-operative diagnosis of PCLs is incorrect even in an experienced, high-volume center [53].

### Worrisome Features and High-risk Stigmata

IPMNs are the most encountered PCL and, in the absence of features associated with a different type of PCL, indeterminate lesions are presumed to be IPMNs. Many studies have investigated features of these lesions that might help guide subsequent management. Since the introduction of the terms “high-risk stigmata” and “worrisome features” in the Sendai guidelines, these features have come to imply varying risks of HGD and/or cancer in definite and presumed IPMNs [54]. The presence of high-risk stigmata implies a greater risk of HGD and/or invasive cancer, ranging from 56%−89% [55]. Studies have found that the risk of malignancy increases in a step-wise fashion with the presence of each additional worrisome feature, from 22%, 34%, or 59% with 1, 2, and 3 worrisome features respectively [56]. Though there is variation in which features are classified as worrisome features and high-risk stigmata between guidelines and what combinations of features warrant further investigation or surgical referral, there are several common threads. These are summarized in Table 1. Significant main-duct dilation, solid, enhancing mural nodules and biliary obstruction are features that warrant additional investigation (i.e. EUS/FNA) and consideration for surgical resection. Mural nodules confer the greatest risk of a cyst harboring malignancy, in one meta-analysis being associated with an OR of 9.3 (CI 5.3–16.1) [57]. The presence of worrisome features should prompt either more frequent surveillance imaging or evaluation with EUS/FNA, though the number of features to needed to recommend EUS utilization varies between guidelines. These features include cyst growth ≥ 2.5 mm/year, main-duct dilation between 5–10 mm, lymphadenopathy, changes in main duct caliber with associated distal (pancreas tail) atrophy, enhancing mural nodules < 5 mm, and cyst size > 3 cm. An elevated CA 19–9 and new-onset or worsening diabetes have also been included as worrisome features [58].

**Table 1:** Recommendations from various guidelines for cessation of surveillance of pancreatic cysts

Guideline	Recommendations for Cessation of Surveillance
IAP 2024	• Individualized assessment of life expectancy and surgical candidacy to inform surveillance recommendations • Cessation of surveillance for small cysts [< 2 cm] without High-Risk Stigmata/Worrisome Features (HRS/WF) after 5 years of stability • Small unchanged BD-IPMN after 5 years of stability • Continued surveillance of these cysts is reasonable given low-quality evidence
ACG 2018	• Individualized assessment of life expectancy and surgical candidacy to inform surveillance recommendations • Continue until age 75, with individualized surveillance recommendations for patients aged 76–85 years
European 2018	• Lifelong follow-up unless limited by life expectancy or surgical candidacy
ACR 2017	• Individualized assessment of life expectancy and surgical candidacy to inform surveillance recommendations • Stop surveillance after 9–10 years of stability without HRS/WF • Stop surveillance for ≥ 80 years [continued short-term surveillance may be appropriate in select cases]
AGA 2015	• Individualized assessment of life expectancy and surgical candidacy to inform surveillance recommendations • Stop surveillance for cysts stable over 5 years

## Guidelines for Managing IPMNs

There are several international and national societies providing guideline recommendations for the management of pancreatic cysts presumed to be IPMNs. Currently, the major guidelines include the 2024 Kyoto guidelines written by the International Association of Pancreatology [59], the 2018 ACG guidelines [60], 2015 AGA guidelines [20], 2018 European Study Group on Cystic Tumors of the Pancreas guidelines [61], and 2017 American College of Radiology guidelines [62]. Here we will provide a summary of recommendations from these guidelines with a specific focus on age-specific recommendations, mostly as they relate to cessation of surveillance (Table 2).

**Table 2.**
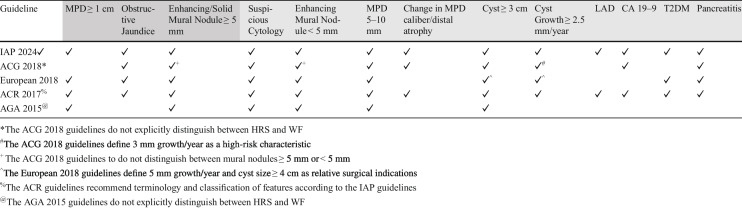
Classification of high-risk stigmata [HRS] and worrisome features [WF] across pertinent guidelines; features in dark grey are generally considered high-risk stigmata and those in light grey are worrisome features

### 2024 International Association of Pancreatology (Kyoto) Guidelines

The IAP guidelines provide recommendations specifically for the management of IPMNs. The 2024 Kyoto guidelines suggest that either a “stop surveillance” or “continue surveillance” approach is reasonable for ‘small unchanged’ BD-IPMN after 5 years given currently conflicting evidence. The guidelines cite two studies underpinning this guidance statement. An international, multicenter study encompassing 3844 BD-IPMN patients demonstrated a risk of incidence pancreatic cancer equivalent to non-cyst-harboring populations for patients ≥ 65 years old with IPMNs ≤ 15 mm and patients ≥ 75 years old with cysts of any size [63]. On the other hand, a Japanese study including 732 patients evaluated incidence rates of pancreatic carcinoma in patients with cysts with up to one worrisome feature at baseline and developing no additional positive features during 5-year surveillance initial diagnosis. They calculated cumulative incidence rates of 3.2% and 9.5% at 10 and 15 years from initial IPMN diagnosis. This study, however, did not stratify patients based on size of IPMN at the end of the first 5 years of surveillance, which appears to be an important prognostic factor. Interestingly, most of these cancers were lesions distinct from the index IPMN. The authors suggest the presence of an IPMN may imply a ‘field defect’ of the pancreas, predisposing these patients to develop pancreatic cancer, which therefore has implications on the need for surveillance [64]. Though the Kyoto guidelines do not make recommendations to discontinue surveillance at any specific age, they do advise discontinuation for patients with a life expectancy of less than 10 years and that providers take a patient’s general condition, comorbidity, and health-related values into account.

### 2018 American College of Gastroenterology Guidelines

The ACG guidelines suggest there is insufficient evidence that surveillance can be discontinued in cysts with 5 years of stability, citing data from a small prospective study including 53 patients who had a 4% risk of cancer after 84 months follow-up [65]. They did, however, also acknowledge the importance of discontinuing surveillance when patients have limited life-expectancy or a comorbidity that makes a patient ineligible for surgical interventions. Recognizing the absence of evidence for making a recommendation, they suggest continuing surveillance in patients until the age of 75 years and designing individualized surveillance plans for patients between 76 and 85 years old.

### 2018 European Guidelines

The 2018 European guidelines suggest that because the risk of progression of IPMN increases over time, these lesions should be surveilled for as long as patient remains a surgical candidate. These recommendations are based on several studies that have documented the development of high-risk stigmata or worrisome features after a 5-year period of stability. In a study conducted by Crippa et al., new worrisome features and high-risk stigmata were found after a median of 71 and 78 months from initial diagnosis, respectively. In 75% of cases, the lesions had no previous stigmata [66]. Furthermore, these guidelines note surgical data reflects an increasing cumulative risk of necessitating surgery over time, rising from 2.9% at 1 year to 72.1% at 10 years, in a cohort of IPMN patients initially devoid of high-risk stigmata [67]. The guidelines also recognize the importance of comorbidities in weighing the risks and benefits of ongoing surveillance given that non-IPMN related mortality is 11 times higher for patients with a Charlson age comorbidity index of 7 or more compared to individuals with lower scores during the initial 3 years of IPMN monitoring [68].

### 2017 American College of Radiology Guidelines

The 2017 ACR guidelines are unique in their provision of different suggested surveillance schedules for patients with incidentally discovered, asymptomatic pancreatic cysts based on age at diagnosis and cyst size [62]. The authors recommend more frequent surveillance for patients with small cysts diagnosed at a younger age, citing evidence suggesting an association between all-cause mortality and incidentally-discovered pancreatic cysts for patients ≤ 65 years old [69]. Specifically, for patients aged 65 to 79 years old with cysts < 1.5 cm, the ACR guidelines recommend surveillance imaging every 2 years for 10 years, given evidence that 90% of cysts do not show significant growth during follow-up. The evidence cited for this recommendation included patients with a median follow-up of only 32 months [70]. Subsequent management and surveillance depends on interval growth of the cyst. For patients < 65 years old, the ACR recommends yearly surveillance for the first five years, followed by every 2 years for an additional 4 years. Surveillance can be discontinued after 9 years of stability or continued based on interval progression during this period. The ACR recommends that if a patient reaches age 80 during surveillance, follow-up should be discontinued, recognizing that some of the follow-up intervals included are based on experiential observations rather than randomized controlled studies. The ACR provides a separate surveillance schedule for patients 80 years old at the time of initial cyst detection. If these patients are deemed appropriate for surveillance based on overall health status, the ACR recommends cessation of surveillance if cysts of any size are stable for 4 years and lack development of high-risk stigmata or worrisome features.

### 2015 American Gastroenterological Association Guidelines

The 2015 AGA guidelines advocate for the discontinuation of surveillance after five years of cyst stability and/or if the patient is no longer considered a surgical candidate. This recommendation was made on the basis of “very low-quality evidence” and no data was cited to evidence the low-risk of progression after 5 years of stability. Based on an overall low risk (0.2%/year) of malignant transformation, the authors speculated that the costs of surveillance and/or surgery would outweigh the benefits of continued surveillance. The 2015 AGA guidelines were also unique in their recommendation to limit further evaluation of PCLs with EUS to only those lesions with at least two high-risk features. Though this was a conditional recommendation on the basis of very low-quality evidence, this recommendation represented a considerable shift away from invasive diagnostic intervention for many cysts.

## Surgical Outcomes in Elderly Patients

Surgical resection of pancreatic cysts generally involves a pancreaticoduodenectomy or distal pancreatectomy with or without splenectomy. Though the Whipple procedure has evolved considerably in the last century, it remains associated with a morbidity of 30–40% and mortality of 3–4% [71]. Though these rates have remained relatively constant, older, more co-morbid patients that would have previously been deemed poor surgical candidates are increasingly undergoing surgery. How these patients fare following minimally invasive and open surgery is an area of active interest. A recently published systematic review and meta-analysis evaluating outcomes in octogenarians undergoing pancreaticoduodenectomy for either benign or malignant indications found that surgery was associated with increased mortality compared to non-octogenarians, higher rates of postoperative complications, and reduced likelihood to undergo adjuvant therapy for pancreatic cancer [72]. These studies highlight the need for careful patient selection when considering the resection of PCLs. While the advent of minimally-invasive and robotic surgical techniques hold the promise of reducing surgical morbidity compared to open surgery, currently available data on the feasibility and benefit of these approaches in elderly patients is conflicting [73, 74].

## New Therapeutic Modalities

Given the morbidity and mortality associated with surgical resection of pancreatic cysts, the development of new, less invasive treatment modalities is of significant interest. One promising new therapeutic modality is EUS-guided pancreatic cyst ablation. Following endoscopic aspiration of cyst contents via FNA, lesions can be lavaged with chemotherapeutic agents (paclitaxel/gemcitabine) with or without 80% ethanol. Many of the adverse effects associated with the procedure (peritonitis, pancreatitis, splanchnic venous thrombosis) have been attributed to the inflammatory effects of alcohol [75]. The small, double-blind, randomized controlled CHARM trial showed that EUS-guided ablation with chemotherapeutics alone was as efficacious with fewer adverse events than combination therapy, providing preliminary evidence that ethanol may not be necessary [75]. Two studies have evaluated the long-term durability of endoscopic ablation. A large, prospective, follow-up study of 164 patients undergoing EUS-guided ablation with alcohol and paclitaxel found that 98.3% of those who achieved initial cyst ablation remained in remission at six-year follow-up [76]. The second study evaluated the efficacy of ethanol and paclitaxel and reported a complete response in 50%, partial response in 25% and a persistent cyst in 25% after a median follow-up of 27 months [77]. However, we have yet to obtain data demonstrating a reduction in the incidence of pancreatic cancer and/or associated mortality with this approach. Where this promising technique fits into the management of PCLs remains an area of ongoing investigation. Though more robust data are needed, it is possible that EUS-guided ablation ultimately offers a more favorable risk–benefit and cost-effectiveness profile compared to surveilling cysts with cross-sectional imaging, surgically resecting them with pancreaticoduodenectomy, or further risk-stratifying them with EUS-FNA and next generation sequencing. Given that IPMNs are presumed to arise within a field-effect that affects the entire pancreas, ongoing surveillance is likely still necessary for IPMNs successfully treated with this modality. However, treatment of MCNs with this modality may be definite, obviating the need for subsequent surveillance.

## Conclusions

There remains significant uncertainty regarding the optimal management strategy of PCLs given the lack of high-quality evidence to guide recommendations. Management decisions for PCLs, be it for surveillance, surgical resection, or invasive diagnostics, should be highly personalized and based on the unique risk–benefit analysis for a given lesion. Especially in elderly populations, physicians should have informed conversations with patients regarding the likelihood of a given PCL to meaningfully affect their quality-of-life or life-expectancy. Given the implications of a pancreatic cancer diagnosis, these can be difficult conversations to have. As the geriatric population in the United States grows, the development of new diagnostic and therapeutic modalities that can improve diagnosis, prognostication and treatment will be of paramount importance to facilitate these conversations.

## Key References

- Marchegiani G, Pollini T, Burelli A, Han Y, Jung HS, Kwon W, et al. Surveillance for Presumed BD-IPMN of the Pancreas: Stability, Size, and Age Identify Targets for Discontinuation. Gastroenterology. 2023 Oct;165[4]:1016–1024.e5.
  - ⚬ Large retrospective study aimed at identifying criteria for safe discontinuation of surveillance in patients with presumed BD-IPMN based on cyst stability, size, and patient age

- Bassi C, Marchegiani G, Giuliani T, Di Gioia A, Andrianello S, Zingaretti CC, et al. Pancreatoduodenectomy at the Verona Pancreas Institute: the Evolution of Indications, Surgical Techniques, and Outcomes: A Retrospective Analysis of 3000 Consecutive Cases. Annals of Surgery. 2022 Dec;276[6]:1029–38.
  - ⚬ A modern review of pancreatic cysts undergoing surgical resection that reviews epidemiologic, clinical, and pathologic characteristics of resected lesions as well as post-surgical outcomes

- Vargas A, Dutta P, Carpenter ES, Machicado JD. Endoscopic Ultrasound-Guided Ablation of Premalignant Pancreatic Cysts and Pancreatic Cancer. Diagnostics [Basel]. 2024 Mar 6;14[5]:564.
  - ⚬ A recent, detailed review of EUS-guided ablation for the treatment of select pancreatic cysts and pancreatic cancer that provides a comprehensive summary of the data on safety and efficacy of this emerging, promising technique

## Data Availability

No datasets were generated or analysed during the current study.
